# Post-Traumatic Nasal Pyramid Reconstruction with a Custom-Made Titanium Implant: A Case Report with Ultrastructural Evaluation

**DOI:** 10.3390/clinpract16100188

**Published:** 2026-10-10

**Authors:** Ioannis Chatzistefanou, Sophia Tsokkou, Sofia Karachrysafi, Kyriaki Papadopoulou, Ioannis Konstantinidis, Alexandros C. Liatsos, Vasiliki Theodorou, Antonia Sioga, Georgia-Alexandra Spyropoulou, Vasileios Petsinis, Theodora Papamitsou

**Affiliations:** 1 University General Hospital “Attikon”, National and Kapodistrian University of Athens, 12462 Athens, Greece; vasipets@yahoo.gr; 2 Laboratory of Histology-Embryology, School of Medicine, Faculty of Health Sciences, Aristotle University of Thessaloniki, 54124 Thessaloniki, Greece; kiki_ppdpl@yahoo.com (K.P.); ikonsc@auth.gr (I.K.); alexliatso@yahoo.gr (A.C.L.); sioga@auth.gr (A.S.); thpapami@auth.gr (T.P.); 3 Research Team “Histologistas”, Interinstitutional Postgraduate Program “Health and Environmental Factors”, Department of Medicine, Faculty of Health Sciences, Aristotle University of Thessaloniki, 54124 Thessaloniki, Greece; sofia_karachrysafi@outlook.com; 4 Department of Neurosurgery, AHEPA University Hospital, Aristotle University of Thessaloniki, 54636 Thessaloniki, Greece; 5 3rd Department of General Surgery, University General Hospital “AHEPA”, Aristotle University of Thessaloniki, 54636 Thessaloniki, Greece; baswtheodorou@hotmail.com; 6 Clinic of Plastic and Reconstructive Surgery, Papageorgiou General Hospital, Aristotle University of Thessaloniki, 56429 Thessaloniki, Greece; gspyropoulou@hotmail.com

**Keywords:** nasal fracture, post-traumatic nasal deformity, nasal pyramid reconstruction, custom-made titanium implant, patient-specific implant, rhinoplasty, transmission electron microscopy, ultrastructure, biocompatibility, IPS implant

## Abstract

**Background/Objectives**: Post-traumatic nasal pyramid reconstruction is challenging in patients with severe deformity and exhausted autologous graft options, particularly after multiple failed surgeries. Custom-made titanium implants may offer precise structural support, but data on their mucosal integration and ultrastructural tissue response in nasal reconstruction are lacking. The aim of this study is to describe what is, to the best of our knowledge, the first reported case of nasal pyramid reconstruction using an IPS (Individual Patient Solutions) custom-made titanium implant and to evaluate peri-implant tissue response with transmission electron microscopy (TEM). **Methods**: A 50-year-old male military personnel with long-standing saddle-nose deformity, nasal obstruction, and prior failed graft-based reconstructions underwent open rhinoplasty with placement of a patient-specific titanium implant designed with three-dimensional virtual surgical planning on the basis of established nasal anthropometric parameters and manufactured as a custom-made device under Regulation (EU) 2017/745. The implant reconstructed the nasal dorsum from nasion to tip and was stabilized with cortical screws; an additional thin titanium plate was used for tip support. Biopsies of nasal mucosa and adjacent connective tissue obtained 24 months postoperatively were processed for TEM to assess epithelial, stromal, vascular, and implant–tissue interface characteristics. **Results**: Clinically, nasal airway patency improved immediately after packing removal, with restoration of nasal contour and stable functional and aesthetic outcomes at 24-month follow-up without complications. Ultra-structurally, the respiratory epithelium showed preserved stratification, intact basal and ciliated cells, normal nuclei and mitochondria, and organized extracellular matrix closely opposed to discrete titanium particles, without granulomatous foreign-body reaction, dense inflammatory infiltrate, or fibrotic encapsulation. Focal ciliary damage, cytoplasmic vacuolization, lamina propria thickening, and vascular elastic lamina remodeling were interpreted as adaptive or transient stress-related changes rather than chronic implant toxicity. **Conclusions**: Custom-made IPS titanium implants can provide stable structural support, satisfactory functional and aesthetic outcomes, and a well-tolerated mucosal interface in complex post-traumatic nasal pyramid reconstruction after failed graft-based surgeries. The ultrastructural findings are being consistent with good local tolerance of the implant at 24 months, showing predominantly preserved epithelial and stromal architecture with only focal, adaptive stress-related changes rather than chronic implant-related toxicity. However, as this is a single case assessed without conventional histology, immunohistochemistry or objective measurement of nasal airflow, these observations cannot establish biocompatibility, efficacy or long-term safety, and require confirmation in larger series with longer follow-up.

## 1. Introduction

### 1.1. Nasal Fracture Reconstruction

Nasal bone fractures are the most common facial skeletal injury, accounting for a significant proportion of trauma-related presentations and affecting both functional and aesthetic outcomes [1,2,3]. Epidemiological data indicate a bimodal age distribution, with the highest incidence predominantly in adolescents and young adults [3,4]. The clinical spectrum of nasal fractures is broad, encompassing simple non-displaced fractures to complex comminuted injuries with septal involvement, each presenting unique diagnostic and therapeutic challenges [2,5,6,7]. Traditional management has centered on closed reduction within two weeks of injury occurrence, which is cost-effective and generally yields satisfactory early results for simple fractures [1,5,6,8]. However, this technique is linked with a substantial rate of secondary deformity, particularly in cases with significant disruption or unfavorable fracture patterns, often necessitating subsequent open interventions [7,8,9]. Open reduction and structural reconstruction are preferred in delayed presentations or when rigid fixation and precise anatomical restoration are required, especially for severe bony deviations, L-strut deformities, and nasal valve compromise [5,8,9,10]. Contemporary systematic reviews and practice management guidelines have sought to standardize the acute and delayed management of these injuries and to reduce the incidence of residual deformity [11].

### 1.2. Personalized Medicine and Advances in the Current Era

Recent advances in personalized medicine and surgical technology have driven the development of custom-made titanium-based implants for structural craniofacial and nasal reconstruction [12,13,14]. Customized titanium-based implants provide the potential for enhanced stability, biocompatibility, and tailored anatomical corrections in complex or revision cases where conventional methods are not suitable [12,15]. The integration of imaging modalities, computer-assisted design, and additive manufacturing enables precise customization that addresses both functional and cosmetic deficits while minimizing the risk of recurrent deformity and airway obstruction [13,14]. Titanium is one of the most extensively studied biomaterials, with a long record of osseointegration and favorable soft-tissue response across dental, orthopedic, and craniofacial applications [16,17]. As the literature increasingly emphasizes individualized, technology-assisted approaches, custom titanium implants represent a promising frontier in the management of challenging nasal bone fractures and post-traumatic nasal pyramid defects.

## 2. Case Presentation

### 2.1. Patient Description

A 50-year-old male patient, military personnel with a history of comminuted nasal bone fractures at an early age, was referred to our clinic at the 424 General Military Hospital, Thessaloniki, Greece, due to nasal obstruction and significant post-traumatic nasal deformity. The patient had undergone prior failed nasal reconstructions in other institutions, utilizing autologous cartilage and bone graft techniques, which unfortunately led to melting and collapse of the nasal bridge, cartilaginous septum, and lateral cartilages.

**Figure 1:**
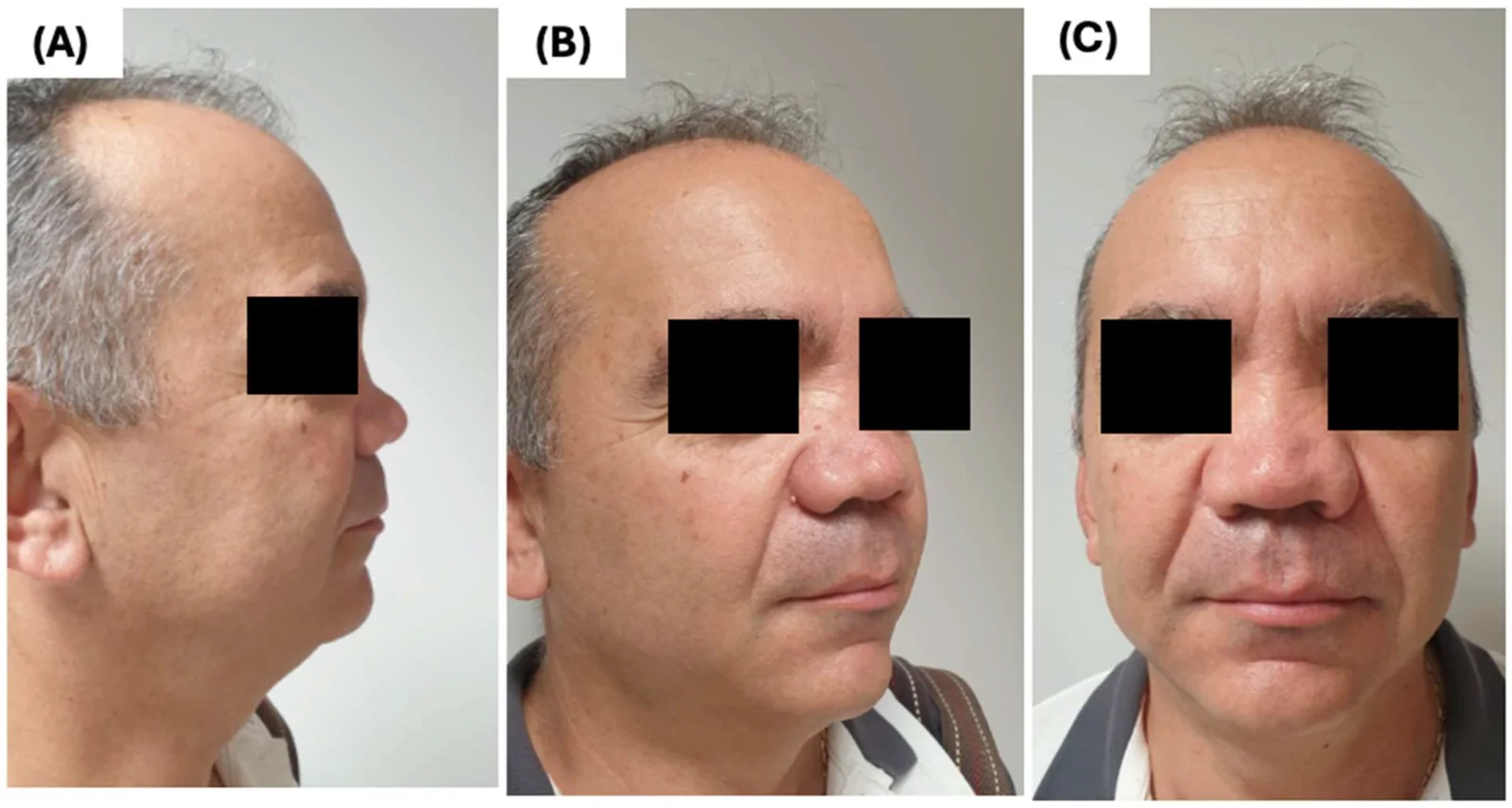
Preoperative documentation. (**A**) Lateral view. (**B**) Oblique View. (**C**) Frontal view. Note the saddle-nose deformity with loss of dorsal height and secondary tip ptosis.

A detailed clinical and three-dimensional (3D) CT evaluation was performed to assess breathing ability and residual nasal anatomy. Nasal obstruction was mainly due to internal nasal valve collapse [10]. Under-reduction of the nasal dorsal hump had led to significant saddle-nose deformity and nasal tip ptosis due to inadequate structural support [18]. The bony nasal septum and piriform apertures were intact. Furthermore, with the patient declining any further bone and cartilage harvests, reconstructive alternatives were very limited. The preoperative clinical appearance and the preoperative skeletal anatomy are documented in Figure 1. Airway assessment was based on clinical examination and nasal endoscopy; objective airflow measurement (active anterior rhinomanometry or peak nasal inspiratory flow) and validated patient-reported instruments such as the NOSE scale were not part of the institutional protocol at the time of treatment, and this limitation is addressed in Section 5.1 [19,20].

We proposed to the patient an innovative solution integrating computer-aided design and a three-dimensional custom-made titanium nasal prosthesis to reconstruct the nasal shape for functional and cosmetic purposes. A reliable reconstruction with a satisfying result was obtained, and the patient resumed his professional activities. No additional surgical procedures were required 24 months post-operatively.

### 2.2. Surgical Planning

Taking into consideration the patient’s surgical history, nasal reconstruction with an IPS (Individual Patient Solutions) titanium-based implant was proposed. Advantages, disadvantages, and possible complications were carefully explained. The patient underwent cone-beam computed tomography (CT) and plain radiographs so that the amount of available bone and cartilage and the appropriate location for the implant could be evaluated. All digital data were transferred to a DICOM (Digital Imaging and Communications in Medicine) file, which was then imported into the IPS CaseDesigner^®^ software version 2.6 (KLS Martin Manufacturing LLC, Jacksonville, FL, USA; available at https://www.klsmartin.com/en/products/individual-patient-solutions-cmf/ips-casedesigner, accessed on 1 February 2026). With the help of 3D virtual surgical planning [13,14], a functionalized patient-specific implant was designed, meeting hard- and soft-tissue anatomical requirements. The material of choice for the implant was titanium, a highly biocompatible material [16,17] that, to the best of our knowledge, had not previously been reported for use in nasal pyramid reconstruction. Fixation was planned with self-drilling cortical titanium screws of 1.5 mm diameter and 5 mm length; screws of 1.2 mm diameter and 7 mm length were held available as an alternative for regions of thinner residual bone but were not required.

#### 2.2.1. Anatomical Parameters Used for Virtual Planning

**Figure 2:**
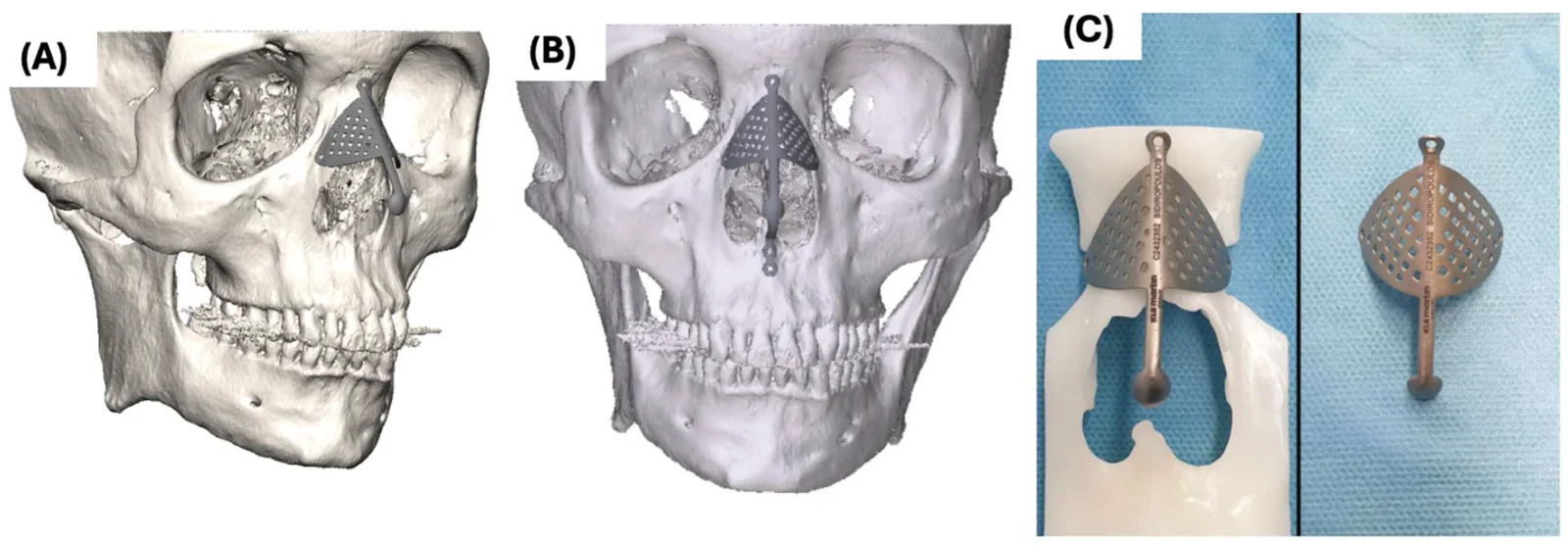
Virtual surgical planning and implant design. (**A**) Lateral view of the planned dorsal profile superimposed on the patient’s skeletal anatomy, showing the reference angles and the planned tip position. (**B**) Frontal view of the computer-aided design of the patient-specific implant. (**C**) Photograph of the manufactured titanium implant before implantation, demonstrating the triangular macroperforated dorsal plate, the curved dorsal strut and the caudal extension for tip support.

The dorsal profile and the tip position were not outlined free-hand. The target profile was defined on the mid-sagittal virtual reconstruction using established anthropometric references for the male nose and was then translated into the implant geometry. The nasofrontal angle was planned within the range of 115–130°, the nasofacial angle within 30–40° relative to the facial plane, and the nasolabial angle within 90–100°, values regarded as aesthetically appropriate in male patients [21]. Nasal tip projection was set according to the Goode ratio, targeting a ratio of 0.55–0.60 between the alar crease–tip distance and the nasion–tip distance, and was cross-checked using the Crumley 3-4-5 right-triangle method, in which projection, nasal height and nasal length approximate a 3:4:5 relationship [21,22]. A straight dorsal aesthetic line with a shallow supratip break was reproduced from the medial brow to the tip-defining points. Dorsal height was referenced to the intact bony septum and piriform apertures, which provided stable skeletal landmarks in the absence of a cartilaginous framework. The resulting virtual profile was reviewed and approved surgically before manufacturing; the planned and the achieved profiles are compared in Figure 2.

#### 2.2.2. Implant Design, Material and Manufacturing

**Figure 3:**
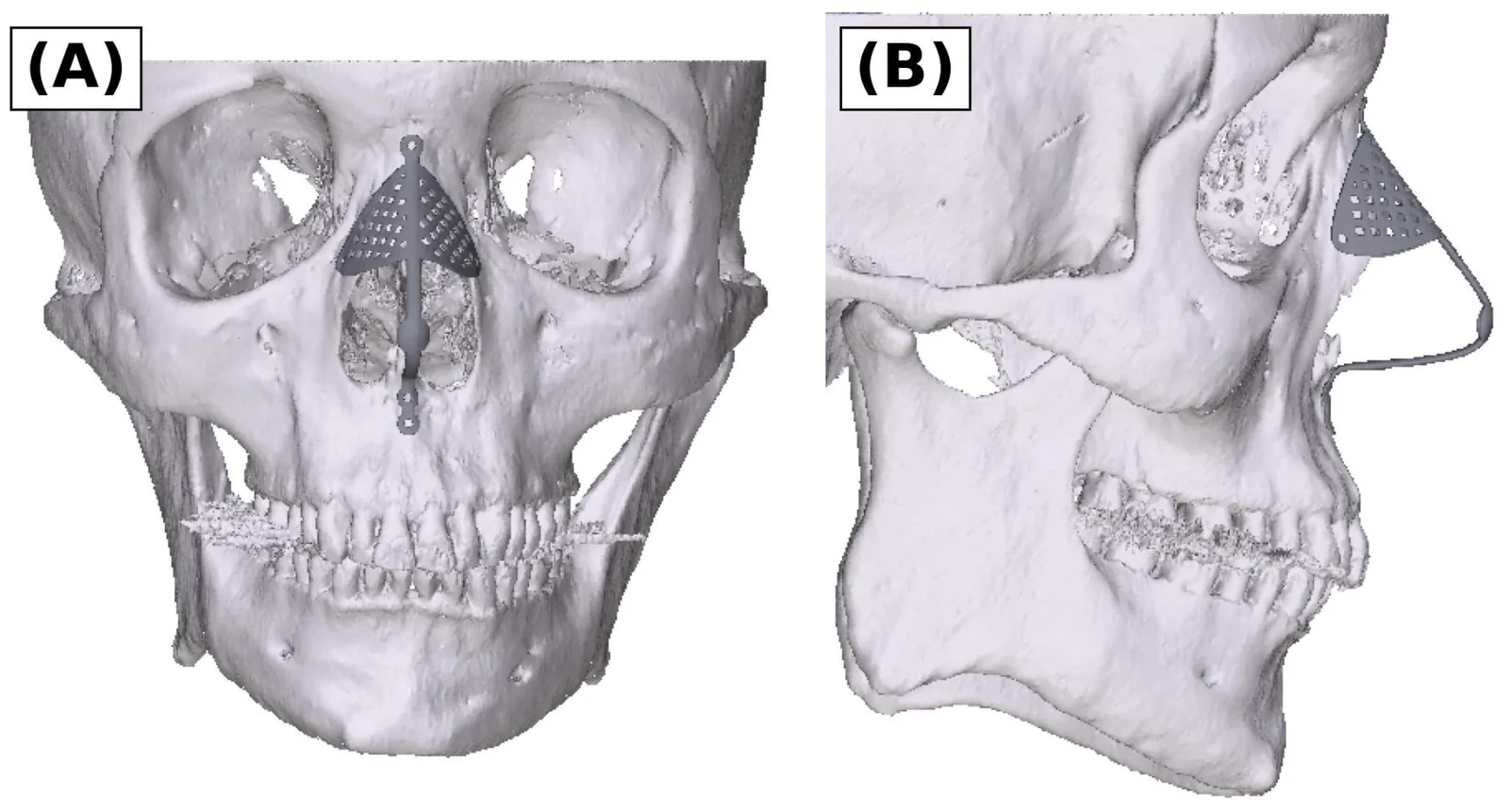
Postoperative computed tomography with three-dimensional reconstruction. (**A**) Frontal aspect, demonstrating appropriate positioning and fixation of the nasal implant. (**B**) Lateral aspect, confirming correct alignment and stable positioning of the nasal implant and of the supporting titanium plate. The macroperforated dorsal plate and the slender caudal strut reproducing the planned dorsal line are clearly visible.

The device consisted of a triangular, macroperforated dorsal plate seated over the nasion and the residual nasal bones, continuing caudally as a slender curved dorsal strut that reproduced the planned dorsal aesthetic line as far as the tip-defining points, and terminating in a caudal extension for tip support (Figure 2 and Figure 3). The perforations were incorporated deliberately: they reduce implant mass and flexural stiffness, they allow fibrovascular ingrowth and mechanical interlocking with the overlying soft-tissue envelope, and they permit drainage of any serous collection beneath the implant. The implant was manufactured from medical-grade titanium alloy conforming to ISO 5832-3/ASTM F136 and was produced by additive manufacturing (selective laser melting), with subsequent finishing of the functional surfaces [23]. The device was supplied in the manufacturer’s standard sandblasted and passivated surface condition, corresponding to a moderately rough topography with a mean roughness (Ra) in the order of 1–2 µm; moderately rough titanium surfaces of this kind are associated with more rapid bone apposition and more stable soft-tissue attachment than machined-smooth surfaces [17,23]. Implant thickness ranged from approximately 0.6 mm at the dorsal plate to approximately 1.0 mm at the caudal strut. The device was cleaned, passivated and steam-sterilised by the manufacturer and delivered ready for implantation.

#### 2.2.3. Regulatory Framework

The implant was supplied as a custom-made device within the meaning of Article 2(3) of Regulation (EU) 2017/745 on medical devices (MDR), having been manufactured to the written prescription of the treating maxillofacial surgeon and intended for the sole use of this individual patient [24]. Conformity was assessed under Article 52(8) and Annex XIII of the MDR. Custom-made devices do not bear the CE marking (Articles 20(1) and 21(1)); instead, the manufacturer draws up the statement required by Annex XIII Section 1, which identifies the patient, and retains the documentation required by Annex XIII Section 2 together with post-market surveillance data for the period prescribed for implantable devices. For a Class IIb implantable custom-made device of this type the involvement of a notified body in the conformity assessment is not required, although the manufacturer’s quality management system is certified to ISO 13485 [25]. Greece has applied the MDR since 26 May 2021, and national market surveillance is exercised by the National Organisation for Medicines (EOF). Beyond compliance with this framework and institutional approval, no separate national authorisation is required for the use of a custom-made device.

### 2.3. Operative Setting

The procedure was performed at the 424 General Military Hospital of Thessaloniki, Greece. Following induction of general anesthesia and endotracheal intubation, an open rhinoplasty was carried out.

### 2.4. Surgical Approach

**Figure 4:**
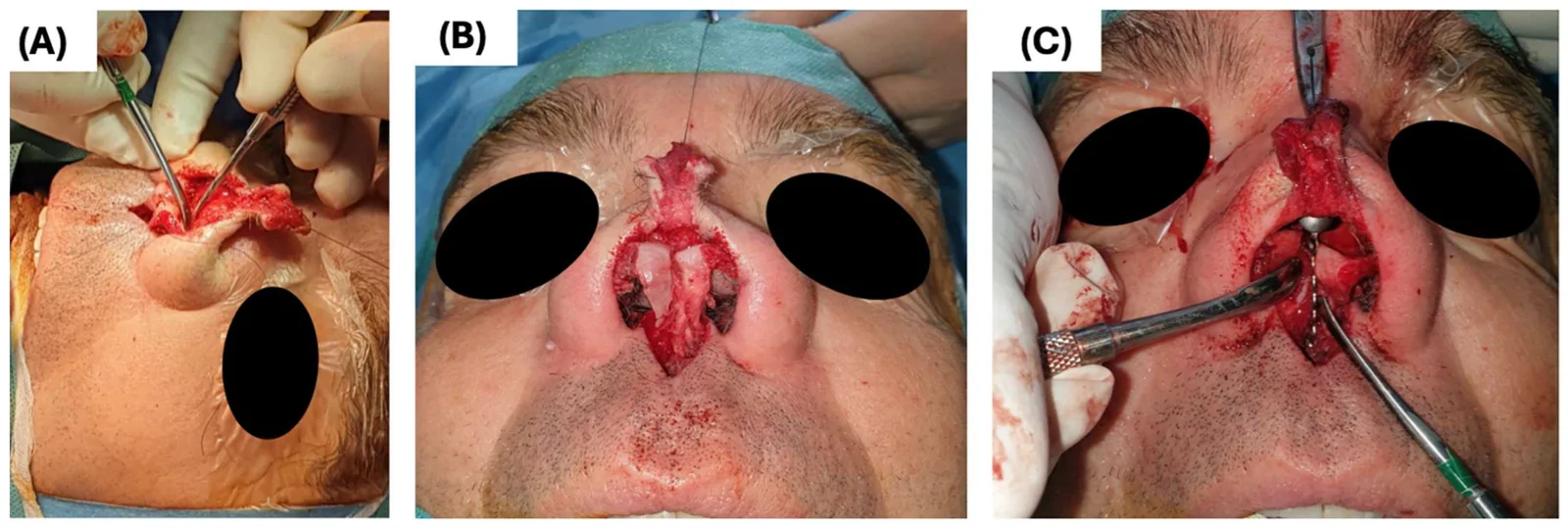
Intraoperative sequence. (**A**) Open rhinoplasty approach through a mid-columellar inverted-V incision extended bilaterally into marginal incisions, allowing exposure of the nasal framework. (**B**) Placement and fixation of the patient-specific nasal implant along the midline, extending from the nasion to the nasal tip and stabilised with 1.5 mm × 5 mm cortical screws anchored to the frontal and residual nasal bones. (**C**) Titanium plate providing additional support to the nasal tip, anchored between the caudal edge of the implant and the anterior caudal surface of the vomer bone.

A mid-columellar inverted-V incision was made, which was then transitioned to marginal incisions with the help of skin hooks and sharp dissection (Figure 4A). The full-thickness mucocutaneous flap was reflected superiorly to expose the bony and cartilaginous remains. The dome and dorsal part of the lower lateral cartilages, the dorsal part of the septal cartilage, and the upper lateral and sesamoid cartilages were missing.

### 2.5. Implant Placement

A periosteal elevator was used to dissect the periosteum over the nasal bones up to the nasofrontal angle. After preparation of the implant hole, the implant was placed in the midline, over the nasal bones, covering the distance between the nasion and the tip, forming an artificial nasal dorsum. Small incisions were made at the level of the inner canthus of the eyes bilaterally to ensure correct implant placement. Cortical screws of 1.5 mm × 5 mm were used to stabilize the implant on the frontal and residual nasal bones (Figure 4B). To achieve adequate support of the tip, a thin titanium plate was used, anchored between the caudal free edge of the implant and the anterior caudal surface of the vomer bone (Figure 4C). A symmetrical small wedge-shaped part of the lower lateral crura was excised bilaterally to create the desired nostril size and base width. Stabilization sutures were placed between the lower lateral cartilages to bring them together and contour the nasal tip. At the end, the mucocutaneous flap was repositioned, and the trans columellar and marginal incisions were closed with interrupted permanent (5-0 nylon) sutures. Adhesive steri-strips were applied horizontally from the nasofrontal angle to the supratip and a larger strip around the infratip lobule, to avoid postoperative edema and support the tip, respectively. A custom-made nasal splint was applied for postoperative stabilization. Finally, sterile petrolatum gauzes were packed in the nasal cavity. A CT scan was obtained postoperatively to ensure the proper position of the nasal implant (Figure 3A,B). No additional soft-tissue interposition graft -such as temporalis or deep temporal fascia, or acellular dermal matrix -was placed over the titanium implant. The implant was covered by the elevated full-thickness mucocutaneous flap, which was replaced so that periosteum, muscle and subcutaneous tissue remained interposed between the metal and the skin. Several measures were taken to reduce the risk of skin thinning, implant show and late extrusion: the dorsal plate was designed to sit flush with the residual nasal bones, its margins were bevelled to avoid a palpable or visible step, and the macroperforated surface was intended to promote fibrous ingrowth and adherence of the overlying envelope. The skin–soft-tissue envelope was thick and well vascularised in this patient, which was considered favourable for coverage. The residual long-term risk is discussed in Section 5.1.

### 2.6. Postoperative Recovery

**Figure 5:**
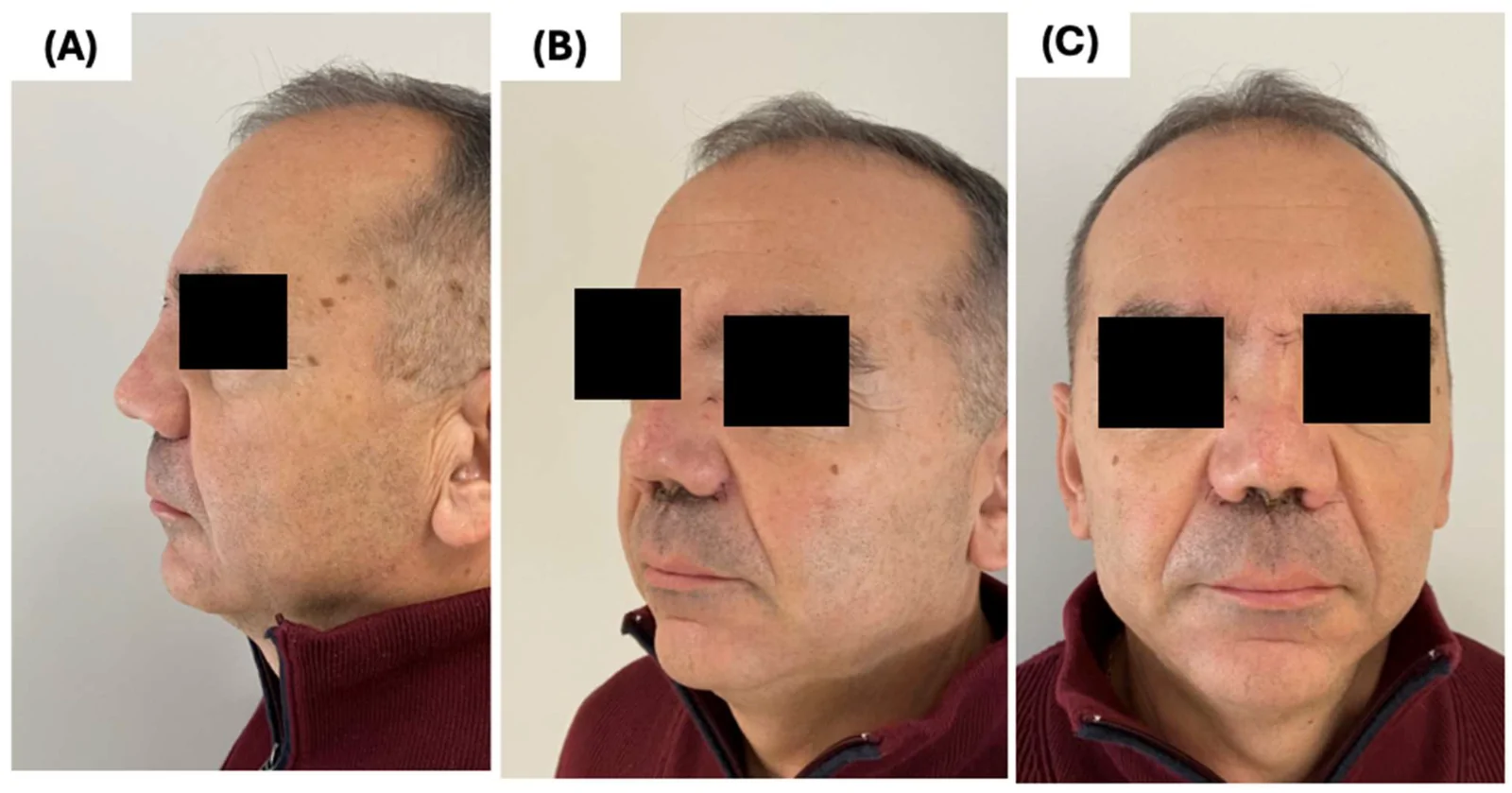
Clinical outcome after reconstruction. (**A**) Lateral view. (**B**) Oblique lateral (profile) view. (**C**) Frontal.

Total hospitalization lasted seven days. Gauzes were removed after five days, while the splint and skin sutures were removed after ten days. As soon as the nasal packing was removed, the patient reported recession of upper airway breathing difficulties and return of normal nasal breathing. Regarding the postoperative cosmetic outcome, the nasal structure was restored without post-traumatic signs, which provided appropriate rehabilitation to improve the patient’s quality of life. At the 6-month follow-up evaluation, during which the patient exhibited no complaints, he was satisfied with the final result. Follow-up was maintained for 24 months, with review at 1, 3, 6, 12 and 24 months. Nasal breathing remained subjectively normal throughout, and the reconstruction remained stable, with no implant mobility, exposure, extrusion, infection, skin thinning or discoloration, and no requirement for revision surgery. The clinical result at 24 months, and its comparison with the virtual plan, are shown in Figure 5. As noted in Section 2.1, no objective airflow measurement or validated symptom score was obtained at either time point.

### 2.7. Tissue Sampling for Ultrastructural Analysis

Biopsy specimens were collected from the nasal mucosa and connective tissue adjacent to the implant site two years (24 months) after placement of the titanium implant. The tissue sample preparation is further explained in Section 3.1. The biopsies were obtained endonasally under local anaesthesia at the 24-month review, from mucosa immediately adjacent to the caudal margin of the implant, with the specific written consent of the patient for this additional procedure. No biopsy was taken at the time of implantation.

## 3. Materials and Methods

### 3.1. Tissue Sample Collection and Preparation for Transmission Electron Microscopy (TEM)

For the tissue sampling, the following procedure was performed:

- The samples were immediately immersed in 2.5% glutaraldehyde in 0.1 M phosphate buffer (pH 7.4) at 4 °C for primary fixation.
- Following rinsing in phosphate buffer, tissues were post-fixed in 1% osmium tetroxide for 1 h to preserve membrane integrity and enhance contrast.
- The specimens were then dehydrated via a graded ethanol series (30%, 50%, 70%, 90%, and 100%) and cleared in propylene oxide.
- For embedding, the samples were infiltrated and embedded in epoxy resin (Epon/Araldite mixture), which was polymerized at 60 °C for 48 h.
- Ultrathin sections (70–90 nm) were cut using a semi-automatic rotary microtome (Leica RM2255, Leica Microsystems, Wetzlar, Germany) equipped with a diamond knife and mounted on copper grids. Sections were stained with uranyl acetate and lead citrate to enhance visualization of subcellular structures.
- The prepared grids were examined by TEM (JEOL JEM-1011 transmission electron microscope (JEOL Ltd., Tokyo, Japan)) operating at 80–120 kV.
- Images were captured digitally at varying magnifications to evaluate cellular and subcellular morphology, including nuclei, mitochondria, microvilli, vacuoles, and connective tissue organization.
- Scale bars were included in all micrographs to provide dimensional references. The scale bar shown in each panel is the primary dimensional reference; the nominal instrument magnification is additionally reported in the figure legend.

### 3.2. Analyses Considered but Not Performed

Conventional histological examination with haematoxylin–eosin staining, and immunohistochemical characterisation of the peri-implant infiltrate (for example CD68 for macrophages, CD3 and CD20 for T- and B-lymphocytes, together with markers of inflammation and fibrosis), were considered during the design of this evaluation but were not performed. The specimen obtained at the 24-month review was deliberately kept small so as not to compromise the mucosal envelope covering the implant, and the entire sample was committed to glutaraldehyde and osmium fixation followed by epoxy embedding, a protocol that is incompatible with routine paraffin histology and with most immunohistochemical procedures. Ultrastructural assessment was given priority because the specific question concerned the state of the epithelium, the basal lamina, the extracellular matrix and the implant–tissue interface at sub-cellular resolution, which light microscopy cannot provide. We accept that the absence of conventional histology and immunohistochemistry materially limits what may be concluded about the inflammatory and fibrotic response, and this is stated explicitly in Section 5.1. In any future series the biopsy will be divided so that paraffin histology, immunohistochemistry and ultrastructural analysis can be performed in parallel on the same tissue.

### 3.3. Ethical Considerations

The study was conducted in accordance with the principles of the Declaration of Helsinki. The patient gave written informed consent for the surgical procedure, for the additional biopsy performed at 24 months, and for the publication of this report together with all accompanying clinical images. A signed copy of the patient consent form has been supplied to the editorial office as confidential material and is not published.

## 4. Results

### Ultrastructural Findings

**Figure 6:**
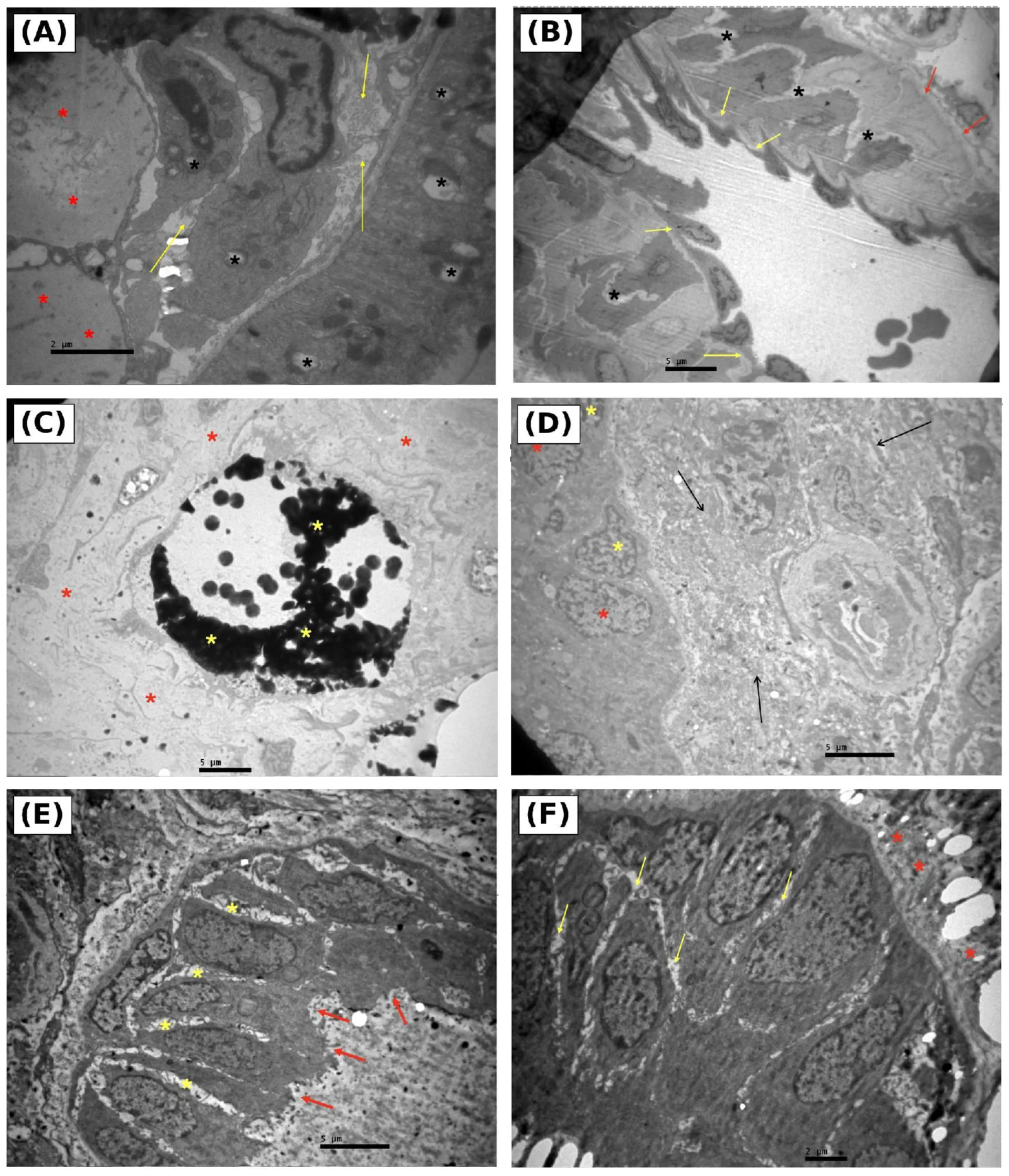
Transmission electron microscopy of the nasal mucosa and adjacent connective tissue obtained 24 months after implantation, in the immediate vicinity of the titanium implant. (**A**) Thickening of the lamina propria (red asterisks) and of the intercellular substance (yellow arrows), with preservation of the architecture of the adjacent epithelium; secondary lysosomes and phagosomes are present within the epithelial cells (black asterisks). Scale bar = 2 µm; nominal magnification ×12,000. (**B**) Section of a small vessel showing increased thickness of the internal (yellow arrows) and external (red arrows) elastic laminae, together with widened intercellular spaces within the vessel wall (black asterisks). Scale bar = 5 µm; ×3000. (**C**) Electron-dense particles of the implant (yellow asterisks), aggregated within a well-delimited space and surrounded by organised extracellular matrix (red asterisks); there is no granulomatous foreign-body reaction, no multinucleated giant cell, no dense inflammatory infiltrate and no fibrotic encapsulation. Scale bar = 5 µm; ×3000. (**D**) Lamina propria without pathological lesions (black arrows); the basal cells (red asterisks) and the ciliated cells (yellow asterisks) of the epithelium show normal architecture. Scale bar = 5 µm; ×4000. (**E**) Respiratory epithelium with focal damage to the cilia at the apical surface of the ciliated columnar cells (red arrows), and intercellular oedema between the epithelial cells (yellow asterisks). Scale bar = 5 µm; ×4000. (**F**) Respiratory epithelium with intercellular oedema (yellow arrows), most marked as widening of the intercellular space at the level of the basal lamina (red asterisks). Scale bar = 2 µm; ×6000.

Transmission electron microscopy of the nasal mucosa and underlying connective tissue adjacent to the IPS custom-made titanium implant demonstrated predominantly preserved tissue architecture with focal adaptive and stress-related changes across all examined fields (Figure 6A–F). In high-magnification views, the lamina propria and intercellular substance appeared thickened, yet the respiratory epithelium maintained an orderly stratification with intact basal and ciliated cells, indicating structural integrity despite local remodeling. Epithelial cells contained numerous secondary lysosomes and phagosomes, reflecting active intracellular processing of extracellular material and suggesting ongoing metabolic adaptation at the peri-implant interface rather than overt cytotoxic damage (Figure 6A,D).

Vascular structures in the vicinity of the implant showed ultrastructural features compatible with remodeling. Sections of small vessels exhibited pronounced thickening of the internal and external elastic laminae, together with expansion of intercellular spaces within the vessel wall, implying altered mechanical load and increased permeability during the healing phase (Figure 6B). These changes were not accompanied by endothelial necrosis or fibrinoid degeneration, supporting a controlled adaptive response rather than vasculitic damage. At the implant–tissue interface, discrete titanium particles were observed embedded within an organized extracellular matrix, with collagen fibers and ground substance closely opposed to the implant surface [26,27]. Notably, there was no evidence of granulomatous foreign-body reaction, dense inflammatory infiltrate, or fibrotic encapsulation, which in this specimen indicates a stable, non-inflamed contact zone (Figure 6C).

Several fields demonstrated completely preserved lamina propria without pathological lesions, and epithelial cells showed normal nuclei, mitochondria, and cell membranes, consistent with sustained cellular viability. In contrast, localized areas displayed features compatible with transient stress: apical ciliary damage or loss in ciliated columnar cells, cytoplasmic vacuolization, and intercellular oedema, most pronounced at the level of the basal lamina (Figure 6D,E). These alterations were focal, did not extend throughout the mucosa, and were not associated with widespread apoptosis or necrosis, suggesting reversible injury related to surgical manipulation or early mechanical loading rather than chronic implant toxicity. Overall, the ultrastructural profile across the examined fields is consistent with a predominantly non-inflammatory, well-tolerated interface between the custom titanium implant and adjacent nasal tissues (Figure 6E,F).

## 5. Discussion

Post-traumatic nasal reconstruction presents unique challenges, especially in cases with prior failed grafts and compromised soft-tissue integrity [9,18]. In this case, which to the best of our knowledge is the first report of nasal pyramid reconstruction using a custom-made titanium implant, we observed favourable clinical outcomes together with ultrastructural findings consistent with local tissue tolerance and adaptation. A claim of primacy in the literature can never be established with certainty; a search of PubMed, Scopus and Web of Science up to August 2026, combining the terms “custom-made”, “patient-specific”, “titanium implant”, “nasal pyramid”, “nasal dorsum” and “rhinoplasty”, retrieved no comparable report, and the claim is phrased accordingly throughout.

The expanded ultrastructural assessment of the nasal mucosa and peri-implant connective tissue is consistent with the clinical impression that the IPS custom-made titanium implant achieved stable integration and a well-tolerated tissue interface in this patient. The coexistence of preserved epithelial and stromal architecture, normal organellar morphology, and organized extracellular matrix deposition around titanium particles is in line with the expected behavior of titanium in craniofacial and nasal applications, where it is known to support long-term tissue compatibility without provoking chronic inflammation [12,16,17,28]. The predominance of secondary lysosomes, scattered vacuoles, and limited connective tissue distortion is more suggestive of physiological turnover and adaptive remodeling than of destructive degeneration, particularly in a setting of complex post-traumatic reconstruction and prior failed grafts.

The vascular changes observed-elastic lamina thickening and increased intercellular spacing-can be interpreted as manifestations of vascular remodeling in response to modified mechanical and hemodynamic conditions created by the implant and reconstructed nasal framework. Importantly, these alterations occurred without ultrastructural hallmarks of vasculitis or thrombosis, which supports their adaptive rather than pathological nature. Similarly, focal ciliary damage, microvilli attenuation, and intercellular edema at the epithelial–stromal interface likely reflect transient insult due to surgical elevation of mucosal flaps, periosteal dissection, and early implant loading—phenomena also described in other titanium implant settings where long-term mucosal and osseous integrity is ultimately preserved [17,26].

The absence of necrosis, extensive apoptosis, granulomatous foreign-body reaction, or dense lymphohistiocytic infiltrates across all examined images is particularly reassuring, as these findings would typically be expected in cases of titanium intolerance, surface contamination, or implant-related infection [27]. Coupled with the patient’s complete functional recovery, lack of infectious or inflammatory complications, and stability of the reconstruction at 24-month follow-up, the ultrastructural data are consistent with a well-tolerated tissue–implant interface in this novel indication, although a single case cannot confirm it. From a reconstructive standpoint, this case raises the possibility that custom-made titanium implants may provide durable structural support and acceptable mucosal compatibility in patients with severe post-traumatic deformity, limited autologous graft reserves, or multiple prior failures [12,15,28]. Future investigations with larger series and integration of ultrastructural analysis, immunohistochemistry, and molecular markers of inflammation and remodeling will be essential to define long-term safety, optimize implant design, and refine patient selection criteria for personalized nasal pyramid reconstruction using titanium implants.

### 5.1. Influence of Implant Design and Surface on Tissue Integration

The physicochemical characteristics of the device are relevant to these findings. Titanium owes its tolerance to a self-repairing surface oxide, predominantly TiO_2_ and a few nanometres thick, which is chemically stable, poorly soluble and readily adsorbs proteins, so that host cells interact with an oxide–protein layer rather than with the metal itself [16,26]. Moderately rough surfaces such as those produced by additive manufacturing and sandblasting increase the effective area available for protein adsorption and cell attachment and are associated with faster and more extensive bone apposition than machined surfaces [17,23]. At the soft-tissue interface, the macroperforations of the dorsal plate allow connective tissue to bridge the implant rather than merely to surround it, converting a smooth foreign-body surface into a scaffold for fibrovascular ingrowth; the organised collagen and ground substance seen in apposition to the implant in Figure 6C are compatible with this behaviour. Conversely, sharp edges, high point loading and thin soft-tissue cover are the recognised drivers of alloplastic implant exposure in the nose [29], which is why the dorsal plate was designed to sit flush with the residual bone and its margins were bevelled.

### 5.2. Titanium Particles at the Implant–Tissue Interface

The discrete electron-dense particles observed at the implant–tissue interface (Figure 6C) deserve specific comment, since their presence might otherwise be read as evidence of implant degradation. Particulate titanium is an expected finding around titanium implants of every kind and does not in itself indicate failure. The recognised sources are abrasion during screw insertion and implant seating, micromotion between implant and bone or between screw head and plate, instrumentation of the metal surface during surgery, and slow passive dissolution of the surface oxide; of these, mechanical release at the time of insertion is quantitatively the most important [27,30]. Several features argue against biological relevance in the present case. The particles were confined to a well-delimited space and were enclosed by organised extracellular matrix rather than dispersed through the tissue. There were no multinucleated giant cells, no epithelioid granuloma, no dense lymphohistiocytic infiltrate and no fibrous capsule, that is, none of the constellation that characterises a clinically significant particle-driven foreign-body reaction. The adjacent epithelium retained normal nuclei, mitochondria and cell junctions, indicating no cytotoxic effect at the concentrations present. Clinically there was no pain, swelling, mucosal discoloration, implant loosening or peri-implant radiolucency at 24 months. This is consistent with the wider implant literature, in which titanium particles are demonstrable around the majority of well-functioning implants while true titanium hypersensitivity remains rare [27,31]. We nevertheless emphasise three caveats. Twenty-four months is short relative to the intended lifetime of the device and particle burden may accumulate. Ultrastructural morphology alone cannot exclude sub-clinical immunological effects, which would require immunohistochemistry to address. Finally, the identity of the deposits was inferred from their electron density and their location rather than demonstrated directly: elemental analysis, for example energy-dispersive X-ray spectroscopy, was not performed and would be necessary to confirm that the particles are titanium.

### 5.3. Limitations

This report is subject to important limitations, which we set out explicitly because they constrain what may legitimately be concluded from it.

First, and most fundamentally, this is a single patient. A case report cannot support generalisation about either the efficacy or the safety of this implant. The findings describe what happened in one man; they do not establish that the technique is effective, nor that it is safe.

Second, the functional outcome was assessed subjectively. No objective measurement of nasal patency was obtained before or after surgery—neither active anterior rhinomanometry, nor acoustic rhinometry, nor peak nasal inspiratory flow—and no validated patient-reported instrument such as the NOSE scale or SNOT-22 was administered [19,20].

Moreover, the biological assessment was restricted to transmission electron microscopy. Conventional haematoxylin–eosin histology and immunohistochemistry for macrophage, T-cell and B-cell markers (CD68, CD3, CD20) and for markers of inflammation and fibrosis were not performed (Section 3.2). Ultrastructure describes morphology within a very small sampled volume: it cannot quantify an inflammatory infiltrate, it cannot phenotype the cells present, and it cannot exclude a focal reaction elsewhere around the implant. The conclusion that the interface is well tolerated is therefore a morphological observation in a limited sample rather than a demonstration of biocompatibility in the immunological or regulatory sense. Sampling bias compounds this, since the biopsy was necessarily taken from mucosa accessible endonasally, which may not represent the tissue in direct contact with the dorsal plate.

Furthermore, follow-up of 24 months is short in relation to the intended service life of a permanent implant. Late extrusion is the principal long-term hazard of alloplastic dorsal implants, and its incidence rises appreciably beyond five years, particularly where soft-tissue cover is thin or has been compromised by previous surgery- precisely the situation in revision cases such as this one [29]. Late infection, implant migration, screw loosening, thermal sensitivity of a metallic dorsum, and the practical consequences of a permanently radio-opaque midfacial implant for future imaging all require longer observation. No conclusion regarding long-term safety can be drawn from two years.

Confirmation of these findings will require prospective series with adequate numbers, objective functional endpoints, paired histological, immunohistochemical and ultrastructural assessment of the same specimen, elemental characterisation of any particulate material, and follow-up measured in years rather than months.

This case not only expands the reconstructive options for patients with limited autologous graft availability but also sets a precedent for integrating advanced imaging modalities into postoperative evaluation. Subsequent research could be enhanced by connecting ultrastructural observations with molecular indicators of tissue integration and stress response, thereby further refining the criteria for implant selection and design.

## 6. Conclusions

In conclusion, the findings of this report indicate that a custom-made titanium implant allowed a satisfactory nasal pyramid reconstruction in one patient with severe post-traumatic deformity and failed prior grafts. The clinical outcome was stable at 24 months, and ultrastructural evaluation showed a predominantly preserved, non-inflamed peri-implant interface. These observations are consistent with good local tolerance; however, arising from a single case with subjective functional assessment, without conventional histology or immunohistochemistry, and with only two years of follow-up, they cannot establish biocompatibility, efficacy or long-term safety. Custom-made titanium implants may nevertheless represent a promising option for challenging nasal reconstructions in which autologous reserves are exhausted, and prospective series with objective endpoints and longer follow-up are required before the approach can be recommended more widely.

## Data Availability

The data presented in this study are available on request from the corresponding authors. The data are not publicly available due to patient privacy restrictions.

## Abbreviations

The following abbreviations are used in this manuscript:

CAD	Computer-Aided Design
CT	Computed Tomography
DICOM	Digital Imaging and Communications in Medicine
IPS	Individual Patient Solutions
TEM	Transmission Electron Microscopy
VSP	Virtual Surgical Planning
